# Impact Assessment of Biological Control-Based Integrated Pest Management in Rice and Maize in the Greater Mekong Subregion

**DOI:** 10.3390/insects10080226

**Published:** 2019-07-30

**Authors:** Dirk Babendreier, Min Wan, Rui Tang, Rui Gu, Justice Tambo, Zhi Liu, Manfred Grossrieder, Monica Kansiime, Anna Wood, Feng Zhang, Dannie Romney

**Affiliations:** 1 CABI Switzerland, Rue des Grillons 1, CH-2800 Delémont, Switzerland; 2 MARA-CABI Joint Laboratory for Bio-Safety, Institute of Plant Protection, Chinese Academy of Agricultural Sciences, Beijing 100193, China; 3 Agricultural Information Institute, Chinese Academy of Agricultural Sciences, Beijing 100081, China; 4 CABI Africa, Nairobi P.O. Box 633-00621, Kenya

**Keywords:** IPM, *Trichogramma*, Greater Mekong Subregion, biological control, integrated pest management, international development

## Abstract

The impact and sustainability of two interventions that have been formulated to introduce integrated pest management (IPM) into rice and maize crops in Southwestern China, Laos, and Myanmar between 2011 and 2016, and were assessed at the end of 2017. From 22 *Trichogramma* rearing facilities established during the interventions, 11 were still producing substantial quantities of biocontrol agents 1.5 years after project support had ended, while seven had stopped operations completely, and four were doing stock rearing only. Through the implementation of biological control-based IPM, slightly higher yields were achieved in maize and rice (4–10%), when compared to control farmers, but the difference was not statistically significant. However, the use of pesticides nearly halved when farmers started using *Trichogramma* egg-cards as a biological control agent. Support from either public or private institutions was found to be important for ensuring the sustainability of *Trichogramma* rearing facilities. Many of the suggested IPM measures were not adopted by smallholder farmers, indicating that the positive impacts of the interventions mostly resulted from the application of *Trichogramma* biological control agents. The following assessment suggests that further promotion of IPM adoption among farmers is needed to upscale the already positive effects of interventions that facilitate reductions in synthetic pesticide use, and the effects on sustainable agricultural production of rice and maize in the target area more generally.

## 1. Introduction

Rice is the most important crop in Southwestern China, Laos, and Myanmar; parts of the Greater Mekong Subregion (GMS). In this region, rice is not only the main source of food, but also provides work and income for 80% of the population [1]. Maize is the second most important cereal crop after rice in the countries mentioned above, produced by around 19 million farmers, and it is a staple for both human consumption and animal feed. Despite significant improvements in rice production in these countries over the past 15 years, productivity has remained low for irrigated rice (i.e., 3–4 t/ha in Laos and Myanmar) [2], while maize grain yields are in the range of 3.6–5.6 t/ha [3,4]. A number of reasons contribute to these relatively low yields, including the impact of pests, diseases, and weeds [2,5]. To date, control measures in the area have predominantly relied on the intensive use of broad-spectrum pesticides, which has led to increased insecticide resistance within the pest population and more frequent outbreaks of secondary pests, such as plant hoppers in rice [6]. Pesticide use in Asia has increased substantially in the last decade, particularly in China, where half of all pesticides applied globally are now used [7]. Looking specifically at rice production in China, the total costs of pesticide use, when including externalities, such as human health and the environment were for example, higher than the US or Germany [7]. More sustainable and economically viable pest management approaches, which are in line with integrated pest management (IPM) practices are, therefore, urgently needed for rice and maize farming in the GMS. Despite being widely accepted as a smart concept for sustainable agriculture, it has often been difficult to put IPM into practice in developing countries as farmers are often pulled between opposing pest management paradigms and different, often justified, interests [8,9]. For instance, agro-dealers may deliver specific pesticide products, or farmers may get subsidies for specific products, which are not in line with IPM.

In both maize and rice, Lepidoptera are key pests. Many major pests from this group have been successfully targeted worldwide by releasing the egg parasitoid *Trichogramma*. In maize, the primary pest in Asia is the Asian corn borer. Large-scale releases of *Trichogramma* are regularly used to control the pest in China [10]. However, for rice, only a few experimental trials exist with no large-scale commercial releases carried out as yet. Prior to the work described herein, no experience of biological control using *Trichogramma* egg parasitoids existed in the target area, and knowledge on IPM was limited to all levels, particularly in the use and production of suitable biological control agents [11].

Therefore, for future pest control options to be successful in the target area, a number of factors had to be considered: They need to present affordable and effective alternatives to the pesticides that have been traditionally used by smallholder rice and maize farmers, and they must be based on simple, low-input technology, that is sustainable and appropriate for local replication. Biological control approaches also need to be resilient to a changing climate, i.e., biological control agents need to be carefully selected for effectiveness at increased temperatures, in order to ensure their long-term effectiveness. Strong institutional capacity and networks need to be in place in the GMS to support and coordinate research, and more critically, to help promote a change in pest management policy and culture in favour of IPM and biological control.

To address these challenges, CABI and the Institute of Plant Protection-Academy of Agricultural Sciences (IPP-CAAS), together with five partners being; Plant Protection Station of Xing’an County, Guangxi Province, China (PPS-CN); Plant Protection and Quarantine Station of Dehong Prefecture, Yunnan Province, China (PPQS-CN); Plant Protection Center, Department of Agriculture, Ministry of Agriculture and Forestry, Laos (PPC-LA); Plant Protection Division, Department of Agriculture, Ministry of Agriculture and Irrigation, Myanmar (PPD-MM); and with additional technical support from the International Rice Research Institute (IRRI) and the biological control agent producer, Tianyi Biological Control Company from Hengshui, China (TBCC), started two projects in 2011 to support smallholder farmers in the GMS to improve livelihoods and food security [11,12]. The key objectives of the interventions were to introduce IPM methods in both crops, which were expected to lead to increased yields, a reduced number of pesticide treatments, reduced input costs, and for maize in particular, generate more business opportunities through enhanced production. As biological control agents for the key rice and maize pests were not previously available in the target area, a major component of the IPM approach was to establish large-scale *Trichogramma* rearing facilities (TRFs) in Laos, Myanmar, and Southwestern China.

While the *Trichogramma* species and production system were mainly developed for the maize IPM project [13], an initial research phase was necessary to develop such a system for the rice IPM project. The most promising *Trichogramma* strains were selected, based on their productivity rates on the rice stem borer target and their suitability for certain climatic factors, including high temperature and intense rainfall [14,15,16]. This research was followed by the development of the most effective, low cost *Trichogramma* production technique, suited to each country and context. For the design and construction of the TRFs, emphasis was placed on using locally available construction materials and techniques, so that they could be maintained and repaired quickly and at low cost. Similar designs were used for rice and maize IPM TRFs, obviously accounting for the needs of the different rearing host (*Sitotroga* versus *Corcyra*). A thorough training programme was set up to ensure staff were in a position to successfully and sustainably rear *Trichogramma* and its host. In addition, business plans and owners’ agreements were developed, which clearly detailed the responsibilities of stakeholders (the TRF management, the owner of the TRF, and the project) with the aim of further enhancing the sustainability of TRFs. 

Different business models operated in the three countries for the two projects to deal with the two different crops. Governmental staff ran the rice TRFs. Whereas for maize, a mixed model was used in China, where the county quarantine station supported TRFs in village buildings and community members were paid a wage. In contrast, in Laos and Myanmar, a village enterprise model was used for maize TRFs, where a participatory, bottom-up approach helped to organise farmer groups and to establish a local implementation group (LIG). Supported through project interventions, the LIG, on behalf of the communities, was the main driver for embedding TRF ownership and operations in a broader village maize marketing concept. Proponents of this concept focussed on opportunities for new market linkages. It was anticipated that existing village or regional structures, e.g., farmer organisations, would be used to run TRFs, while other community members owned small side businesses (maize processing, savings through coordinated purchase of maize farming inputs, joint product marketing for better prices) that would contribute to the necessary start-up costs of the TRFs, which would in turn improve maize yields once operational. To support this innovative approach, farmers and LIG members were not only trained in practical rearing techniques and facility-related aspects, but also in business skills, and they received support to reach out to local marketing consultants. 

For both maize and rice, the idea was that, in the longer term, the costs of production would be covered by charging farmers for egg-cards, so that the TRFs could become self-sustainable. In mid-2016, as the programme was nearing its end, 12 TRFs had been set up within the rice IPM project and 10 within the maize IPM project—four each in China and Myanmar, and two in Laos. However, the production and delivery of biological control agents was not an easy task, particularly in Laos and Myanmar, where it is a completely new concept. Production of *Trichogramma* egg-cards collapsed a few times at the beginning, which meant relatively low amounts were produced by the end of 2015. In contrast, up to 85 g of *Corcyra* eggs (enough to cover about 18 ha, when parasitized by *Trichogramma*) were produced daily in each of the TRFs situated in Yunnan and Guangxi provinces, China, during 2015.

In addition to the production and delivery of *Trichogramma*, an IPM strategy for rice was jointly developed in each of the target countries, outlining the relevant IPM measures recommended for farmers to implement in their fields. The strategy included general IPM practices, such as balanced fertilisation, pest monitoring, alternative wetting and drying, and the growing of flowering plants on the bunds, as well as the application of the *Trichogramma* wasps. Finally, farmers received training on the application of biological control agents and other IPM measures. In the rice IPM project, a total of 137 training sessions, reaching more than 6400 farmers, were provided in the target countries. Approximately 10,000 maize farmers were targeted through an information campaign via radio, focusing on the IPM of major pests in maize storage.

The aim of the study presented here was to assess the impact of the rice and maize interventions described above. It was assumed that within the relatively short time between the end of the interventions and the impact assessment (i.e., 18 months), large-scale effects would not be expected. Instead, the following research questions were addressed:

1. To what extent are the TRFs under each business model still operational, and why or why not? (supply chain aspects related to delivery of the *Trichogramma*);
2. What are farmers’ opinions/views on the use and effectiveness of *Trichogramma*?
3. What changes can be observed for other stakeholders, such as local authorities, local extension workers, and country project implementation teams?

A lesson-learning approach was taken to understand causal pathways, as well as the outcomes and impacts of the interventions. This was seen as particularly relevant and insightful because the two projects followed somewhat different approaches. 

## 2. Materials and Methods 

### 2.1. Study Areas and Sampling Strategy

The intervention outcomes were assessed in all three target implementation countries during October–December 2017. The assessment period came just after the 2017 rice and maize growing seasons, and 18 months after project funding stopped. To investigate the rearing activities and outputs of the 22 TRFs, a pre-survey interview was conducted with the project managers in the three countries via e-mails, phone calls, or face-to-face meetings in June-July 2017. Eleven representative TRFs were selected for on-site visits to collect information, in order to assess the projects’ outcomes. Staff from two more TRFs were invited to one of the selected TRF locations to obtain additional information. In total, information was collected from three TRFs in Laos, three in Myanmar, and seven in China (see Table 1). In each country, at least one rice TRF and one maize TRF was selected, and the selection included both TRFs with high egg-card production, as well as TRFs that had apparently stopped operation. 

The TRF sites were seen as key sites of the intervention, since the IPM training, the *Trichogramma* releases, and other project activities were implemented in the vicinity of the TRFs. Key informant interviews (KIIs), focus group discussions (FGDs), and farm household questionnaire surveys (HHS) were carried out to collect information in the selected TRF areas for assessment of the projects’ outcomes. However, the latter were only implemented in Southwestern China, where a substantial amount of the *Trichogramma* was produced and delivered to farmers.

#### 2.1.1. Farmer Focus Group Discussions (FGDs)

One to four FGDs were carried out in at least one maize and one rice site per country. Each of these FGDs was attended by about 10 rice or maize farmers. At least one group of farmers, that were involved in each of the FGDs, had applied *Trichogramma* egg-cards in 2017 (the treatment group), and one group had not applied the egg-cards in 2017 (the control group). Emphasis was placed on matching all other factors of the treatment and control group farmers. A total of 17 FGDs were conducted in China, Laos, and Myanmar, involving 69 maize and 116 rice farmers (see Table 1). The main questions asked during the FGDs dealt with the effect of the interventions on farmers’ fields, including: What benefits did farmers observe in their field compared with the conventional pest control methods they adopted before? Did farmers understand and follow the *Trichogramma* release approach they received training on during implementation of the projects? Did farmers follow some of the IPM practices introduced by the projects and what was the adoption rate? Were farmers willing to buy *Trichogramma* egg-cards if they became available on the market?

#### 2.1.2. Key Informant Interviews (KII)

The seven different types of key informants included, staff from the country project implementation team; local agricultural authorities; local IPM trainers or governmental extension staff; TRF managers and staff; village committee members or village heads in the *Trichogramma* release area; TRF customers, and agro-dealers. Wherever possible, interviews were implemented with representatives from all seven groups of key informants. KIIs were conducted for all 13 selected TRFs, and a total of 61 KIIs were carried out across the three target countries (Table 2). In China, all KIIs were conducted by an independent impact assessment team member, while in Laos and Myanmar, the country project managers acted as interpreters for some KIIs. The questions were primarily aimed at establishing the sustainability of TRF production in view of the *Trichogramma* egg-card supply chain. Some of these questions included: What is the local market for *Trichogramma* egg-cards? Which elements of the TRF business plan worked well and which did not? What are the advantages and challenges for the TRFs established by the projects? When do the TRFs plan to commercialise and run sustainably? What was the main reason for stopping operations (for those TRFs that were no longer producing egg-cards)? What is the business model going forward (for those TRFs still running)? What benefits did the TRFs gain from the projects?

#### 2.1.3. Farmer Householder Questionnaire Survey (HHS)

For a subset of selected TRFs in two regions in China, for which substantial production and the release of egg-cards was ongoing in 2017—Xing’an county, Guangxi Province and Dehong prefecture, Yunnan Province—a HHS was conducted. The main treatment factor was the application of *Trichogramma* in farmer rice or maize fields, thus, we selected farmers that applied *Trichogramma* egg-cards in 2017, and compared their feedback with farmers not using egg-cards in their rice and maize fields. Farmers were selected at random from villages in which *Trichogramma* had been provided, and villages in the same county with similar agroecological conditions, where *Trichogramma* had not been delivered. Three survey teams were established, each with 5-8 enumerators (local extension workers). Before the field survey was carried out, a one-day training was implemented to ensure enumerators fully understood all of the questions in the questionnaire and to provide an opportunity for them to practice asking the questions in pairs. Finally, to ensure the completeness and logicality of answers to the questionnaires, a self-check by enumerator, an exchange-check by other enumerators, and a final-check by the team leader were implemented. A total of 195 farmer household surveys were completed in China, where all farmers were asked for permission to conduct the interview beforehand. For the rice project, 105 treatment and 30 control farmers were interviewed, while 50 treatment and 10 control farmers from the maize project were interviewed (see Table 1). Differences between the treatment group and control group were tested using *t*-tests or chi^2^-tests in Stata software. Notably, we would like to stress that data were based on farmers’ perceptions and could not be verified in the field.

## 3. Results

### 3.1. Supply Chain Aspects Related to Delivery of the Trichogramma

The KIIs revealed that, among a total of 22 TRFs established in the three target countries during the project period, 20 TRFs (12 for the rice IPM project and 8 for the maize IPM project) were running in mid-2016, when both projects had just finished (see Table 3). By the end of 2017, based on the findings of the KIIs and on-site visits, 11 TRFs (eight for the rice IPM project and three for the maize IPM project) were still producing *Trichogramma* egg-cards. Meanwhile, four TRFs had continued stock rearing to maintain production in the event of a future need (see Table 3 for details). Five TRFs had stopped operating completely between the end of the projects and the time of the assessment. 

To support the sustainable operations of the TRFs, business plans and owner agreements (setting out partner responsibilities) were developed in both projects. However, the assessment found that these documents only partially worked for those TRFs still operating in 2017. The 11 TRFs still producing *Trichogramma* egg-cards followed their production plan, which was a relevant component of the business plan, but other components, such as the *Trichogramma* marketing plan, have not been implemented. Most TRF managers were barely aware of the business plan or owner agreement. 

For the two maize TRFs still running in 2017 in Laos and Myanmar, the community-based local implementation and producer groups still played a key role in TRF operations. However, only the TRFs in Laos continued egg-card production in 2017 for mass release, and in this producer group, two out of the three members were district level governmental extension officers. This indicates that, in this case, the producer group is not a purely village community-based group. 

Furthermore, in the maize IPM project, some innovative community-based approaches for long-term sustainability were piloted in one TRF in Laos and three TRFs in Myanmar. The approaches aimed to generate profits and possibly support the operations of the TRFs, as well as strengthen market linkages for maize farmers. For instance, a sweet maize processing room was established in Sanakham, Laos, while a maize post-harvest information centre was established in Pinphit, Myanmar, and marketing centres were established in each of Sakhanthar and Kharshi, Myanmar. The on-site visits revealed that these small innovation projects, managed by rural communities, are still running well and they had created some income for the farmer groups. However, the income was used to further scale up these business activities, rather than to support TRF operations, as farmers envisaged more benefits from the former.

The main reason that TRFs stopped *Trichogramma* egg-card production was a shortage of stable funds to cover the running costs of TRFs, i.e., the costs of water and electricity, rearing materials, and staff salaries. Rearing technique was less of an issue for almost all TRFs, except for one maize TRF in Myanmar, which was run by a farmer group without any technical support from the local governmental extension in 2017. This TRF failed to mass produce the host, *Sitotroga*, when they changed the rearing media from maize to rice in order to save money.

The study found that three TRFs were selling cards to customers in 2017, including two rice TRFs in China and one rice TRF in Myanmar. However, the customers of the two rice TRFs in China were governmental agencies in neighbouring counties, who bought egg-cards (using funds, such as the ‘Green Control’ or ‘Pesticide Reduction Programme’) and then distributed the cards to local farmers free of charge. The Yangon TRF in Myanmar is the only TRF where local farmers paid for egg-cards. The farmers paid 50 kyat/card, which equals $3.7 (US) for a release of 100 egg-cards on one hectare. The price was calculated without charging for the facility staff salaries or water and electricity costs, indicating that, even in this case, the TRFs could not run commercially, based on this low egg-card price. The relatively low price of egg-cards was facilitated by local government investment, and the attitudes among plant protection officers that the use and dissemination of locally produced biological control agents should be supported by government funding. In fact, biological control products, even when delivered by commercial companies, are generally given away for free by the government or heavily subsidised in Myanmar and China. KII’s further indicated that competitive prices could only be achieved with more modern technology and infrastructure. However, like many development interventions, the projects focused on low-cost approaches to IPM and *Trichogramma* rearing, using local materials. 

### 3.2. Farm-Level Impacts Related to Adoption of Trichogramma 

In the HHS carried out in two regions in China, treatment farmers, who applied *Trichogramma* egg-cards to their fields, and control farmers, who did not, were similar in terms of household size, household education level, and involvement in farming activities (see Table 4). Treatment farmers owned and cultivated significantly more land than control farmers, but they did not vary significantly in terms of areas allocated to rice or maize production.

When comparing the yields reported from the last harvest in 2017 between treatment and control farmers, the average yield of rice and maize tended to be slightly higher for treatment farmers, however, this trend was not significant for either crop (see Table 5). While virtually all rice farmers applied pesticides to their crop, only about two-thirds of maize farmers were doing so, with no difference between treatment and control farmers. However, significant differences between the treatment and control groups were found for the proportion of households reducing the amount of pesticides applied (see Table 5), at least for rice farmers. Although, these yields have not been not verified and the results are based on farmer perceptions.

**Figure 1:**
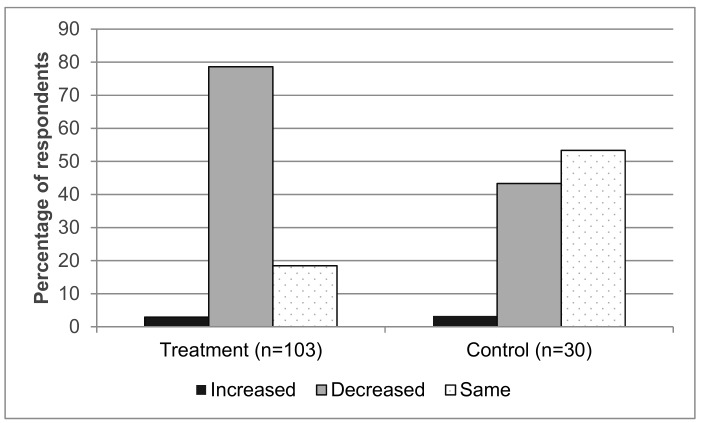
Trend in use of pesticides between 2015 and 2017 for rice farmers in the project implementation area in Southwestern China, based on farmers responses to the household questionnaire surveys (HHS) (Chi^2^ = 14.9, *p* < 0.001).

Similarly, a significant reduction in the use of pesticides on rice was seen between 2015 and 2017 for IPM rice (treatment) farmers, compared to the control farmers (Table 5). A detailed analysis revealed that most of the treatment farmers reported a decrease in pesticide use, while the majority of the control farmers reported no change in pesticide use between 2015 and 2017 (see Figure 1). Interestingly, and considering both, rice and maize farmers, there is no significant relationship between yield and cost spent on pesticide per hectare, neither for treatment farmers (*r* = −0.020, *P* = 0.82, n = 155) nor for control farmers (*r* = −0.017, *P* = 0.92, n = 40), indicating that higher pesticide inputs do not translate to higher yields.

Farmers who reported a reduction in the amount spent on chemicals were also asked for the potential reasons for this reduction (see Table 6). According to their responses, the adoption of alternative IPM methods and good pest control levels were the main drivers of reductions in the amount spent on chemicals among the sampled households. Significantly fewer treatment farmers reported pest and disease outbreaks compared to control farmers (see Table 6).

Based on treatment farmers’ reports before, and after, applying *Trichogramma* egg-cards, there was a significant decrease (41–47%) in the number of times insecticides were used after the egg-cards were applied, for both rice and maize farmers (see Table 7). Correspondingly, after the application of egg-cards, there was a significant decrease in the costs incurred from purchasing insecticides by 35–40%, for both maize and rice farmers (see Table 7). 

Corroborating the above results, most treatment farmers reported an increase in yield and a decrease in the number of pesticide spray applications. During the FGDs, 45–100% of maize farmers reported a crop yield increase whilst applying *Trichogramma* releases. At the same time, reductions in the number of pesticide sprays were reported by 0–30% of maize farmers involved in the FGDs, with chemical costs decreasing for 0–35% of this group. For rice farmers, 0–46% reported an increase in yield after applying *Trichogramma* egg-cards, while 29–35% of farmers surveyed in the FGDs reported a reduction in chemicals applied. Agricultural input costs decreased for 0–65% of rice farmers. As expected, the *Trichogramma* releases were the main reason mentioned by farmers for the decrease in pesticide treatments, together with the availability of other alternative plant protection measures, such as pheromone traps.

**Figure 2:**
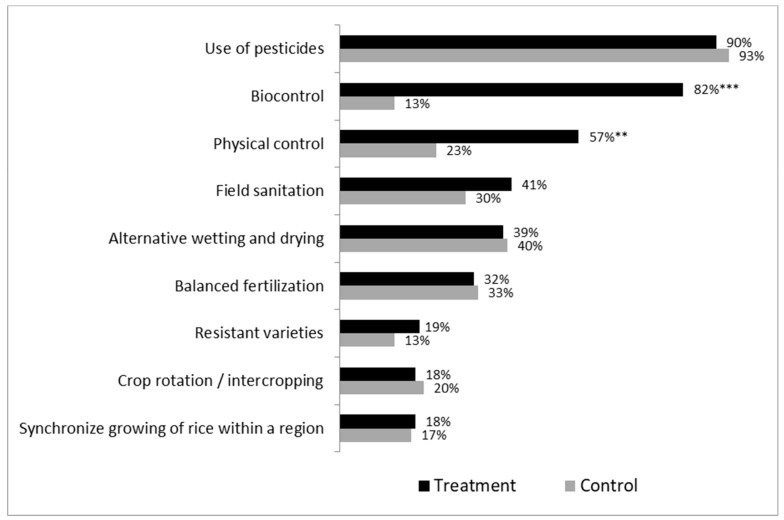
Adoption of rice IPM practices as reported by farmers during the HHS in the last cropping season of 2017 (treatment n = 105; control n = 30). Note: *** and ** indicate that there are significant differences between treatment and control farmers, based on *t*-tests, at the 0.1% and 1% significance level, respectively.

According to feedback from farmers and local extension workers (during KIIs, and the FGDs), farmers involved in the rice IPM project not only learnt about the release of *Trichogramma*, but also about many other IPM methods, including water/fertiliser management, pest monitoring, etc. In fact, data from the HHS confirms that, significantly more treatment than control rice farmers applied physical control (mostly traps) and field sanitation practices (see Figure 2). 

However, no differences were observed between treatment and control groups in the implementation of a number of additional IPM measures recommended during the project, such as balanced fertilisation, or alternative wetting and drying (Figure 2). For both, rice and maize IPM projects, most treatment farmers implemented a biological control, while very few control farmers did so.

Similarly, farmers in 10 out of 17 FGD groups said that they would like to buy *Trichogramma* cards. The study found that 100% of the maize treatment farmers in China expressed their willingness to buy *Trichogramma* cards, with expected prices of around $0.4 to $0.56 per card (100 cards needed per ha). A total of 75% of rice farmers involved in the FGDs in China expressed their interest in buying the egg-cards, but at lower prices, ranging from $0.08 to $0.31 per card. In Laos, 80% of treatment farmers expressed their interest in buying cards at 0.12 $ per card. In Myanmar, 25% of rice farmers involved in the FGDs said that they would like to buy egg-cards at a price of $0.04 per card, while others were not yet confident of the effects of *Trichogramma* releases in the fields and thus not interested to pay for it. A common statement was that farmers are interested in buying cards, as long as the price was lower than what they usually pay for chemicals. For one TRF in China (Hejiatang), which produced egg cards to cover 200 ha of rice fields in 2016, we estimated the costs of production (assuming no costs for the building) on a yearly basis as follows: Salaries - $6000; electricity and water - $700; inputs for diet (rice bran etc.) and egg cards - $1200; depreciation costs for equipment (10% annually) - $1000, i.e., a total of $8900. Based on these figures, costs per release and per card would be in the range of $0.45, and would therefore be in the range of what farmers indicated they would likely be willing to pay.

### 3.3. Behaviour and Knowledge Change Among Other Stakeholders

In all three target countries, knowledge and behaviour changes of other stakeholders, besides farmers, were reported in the KIIs, including changes among the country project implementation team, local agricultural authorities, local extension workers, and TRF managers. In 2017, the two IPM projects won the second place Science and Technology award of Dehong Prefecture, Yunnan Province, China, due to their achievements regarding pesticide reductions in the project implementation areas and the manifold spill-over effects to the whole prefecture. 

Improvements in biological control knowledge, based on IPM, were widely reported by all stakeholders interviewed, particularly the local governmental extension workers, who were personally involved in the training of trainers (TOTs), and farmer training organised by the projects. In Sanakham District in Laos, this was the first time that extension workers received training on IPM and biological control. In other project implementation areas, although IPM was not a new concept, attending TOTs significantly improved the IPM and biological control knowledge of extension workers. The key IPM components, such as preventative cultural control methods, pest monitoring, and non-chemical control methods, including biological control, became a regular part of the extension workers’ daily work.

All country partners indicated that they became more confident about promoting biological control to farmers after the two projects were implemented. It was agreed that, with the establishment of TRFs, the biological control was no longer just a theoretical concept. The TRFs established by the projects have received a high profile, and repeated visits and inquiries from farmers, universities, and plant protection organisations outside the designated coverage areas, in all three countries. 

## 4. Discussion

Impact studies have been carried out several times for IPM-related interventions; however, these were often focused on IPM farmer field schools (FFS) e.g., [17,18,19]. IPM interventions where farmer training was not the main component are less often assessed (for an exception see [20]). In many cases, studies of FFSs have shown positive outcomes, for example, rice farmers in Sri Lanka drastically reduced insecticide use compared to control farmers, over five years after attending a FFS [21]. A study in Indonesia showed that an FFS had no effect on pesticide use and rice yields eight years after training. It is one of the few studies that did not demonstrate the positive impacts of FFSs [22]. However, it is likely that studies, such as the latter, are underestimating the effects of FFSs because knowledge is often spread from farmers involved in FFS to those not directly involved, increasing its general effects over time. This, however, should be of minor importance in the present study because an impact assessment was conducted only 1.5 years after the intervention came to an end. 

The impact of IPM interventions can be assessed in many ways. Here the focus was on short-term impacts, i.e., a reduction in pesticide use, an increase in yields, or improved pest management. In addition, the sustainability of the production and use of the biological control agent was considered. As such, the interventions evaluated here met the needs of all target countries regarding their agricultural development strategies, including the ‘Green Control and Professional Unified Control’ and ‘Pesticide and Fertilizer Zero Growth Action Plan (2015–2020)’ in China, and the ‘Clean Agriculture’ programme in Laos. This alignment with national agricultural development strategies was highlighted repeatedly by the national and local agricultural authorities involved in the projects. The IPM principle is a key component in all of these recent national policies/programmes, which helped to create a conducive policy environment for the maize and rice IPM projects in the target region.

Nevertheless, the introduction of biological control-based IPM in Southwestern China, and particularly in Laos and Myanmar, was not easy, as this was a novel approach for farmers. Difficulties were envisaged, both on the production side, as well as in farmer uptake. The former point refers to problems with producing high amounts of biological control agents at low prices, while the latter refers to farmers being unsure about the effectiveness of biological control agents, as well as their limited willingness to spend money on it. As a result, the commercially sustainable operation of TRFs has not been realised at the time of this assessment, i.e., 18 months after funding of the projects finished. From information gathered during the KIIs, the reasons for not achieving sustainability were found to be slightly different in the three countries. In China, when compared to other natural enemy rearing facilities in the same region, the TRFs established by the two projects have very basic and low-cost equipment, with a lower level of automation, resulting in a relatively small production capacity overall. Still, taking one rice TRF in Xing’an as example, the TRFs were able to produce *Trichogramma* egg cards at about 0.45 USD per card, or 45 USD per hectare, thus at the higher end, but within the range of what farmers might be willing to invest, based on reports from this survey. However, in addition to issues mentioned above, many non-chemical pest control products (biological control agents, light traps, etc.) were recently distributed free of charge to farmers with support from governments, in order to meet their targets on pesticide reduction. It is, therefore, likely that there were very few farmers, especially smallholder farmers—the main targets of rice and maize IPM projects—who really need to use their own money to buy such alternative pest control products. Thus, even though the free delivery of environmentally benign products by governmental bodies is positive for the short-term reduction of chemical use, it may add another level of difficulty for commercial suppliers of biological control agents, who are trying to become profit-making entities.

In Myanmar and Laos, agricultural production is less intensive than in China, and most maize and rice farmers do not regularly apply insecticides to these staple crops. Although, this opens up an opportunity for farmers to start using biological control products, such as *Trichogramma* egg-cards instead of insecticides, it is also more difficult to persuade farmers to invest in such inputs because most farmers have not previously had any pest management costs. In addition, *Trichogramma* cards are not as effective when they are only used by individual smallholder farmers on their fields, which are often in the range of one hectare or below, due to the dispersal of the biological control agent. To reduce the dispersal of *Trichogramma* wasps from release fields, the two projects were aimed at implementing a village approach, where ideally, all of the farmers in a particular village used the biological control agent in their fields. This, however, was not fully achieved in all cases, which supports the observation reported by Parsa et al. [9], that the requirement of collective action by farming communities is one of the most important obstacles in the implementation of IPM. 

One reason that partners in Laos and Myanmar struggled to get high outputs from the TRFs was linked to the climatic conditions in these countries. The hot and humid climate made it challenging to separate the rearing rooms from the outdoor environment, and resulted in high incidences of pests in the TRF, such as *Habrobracon* parasitoids attacking the *Corcyra* larvae. In conclusion, national partners in Myanmar stated that, even though rice farmers paid a small amount of money for *Trichogramma* egg-cards, it was not a feasible development strategy to have fully commercial TRFs in Myanmar. Thus, when the project finished, government funding was obligatory for the continued operation of TRFs in Myanmar and the situation in Laos was found to be very similar. 

From the KIIs, it became clear that the strategy-type documents, developed within both projects, had been of limited value for TRF operation. For instance, owner agreements detailing the responsibilities of respective stakeholders and business plans were hardly followed. In contrast, detailed manuals (e.g., on biological control agent production) were highly appreciated and used by TRF staff. The commitment of TRF staff and other key implementing partners was very important in achieving the projects’ aims.

For the 11 TRFs still producing egg-cards, the operational funds in 2017 were all from governments, regardless of the country, and whether the TRFs were established in the rice or maize projects. Comparing the two projects, a somewhat higher proportion of rice than maize TRFs were still in production (67% versus 38%). While TRFs in the maize project were established through the LIGs, with community-based farmer ownership and management, TRFs in the rice project were more affiliated with the national partners, i.e., governmental agencies linked to plant protection. Due to the close link with national partners, the rice TRFs had better access to governmental resources when the projects finished. For the three maize TRFs, which were still producing egg-cards during this assessment, financial support was provided by governmental funds from national partners.

Despite the dependence on governmental resources mentioned above, in all three target countries, this dependency was reduced to give more control and ownership over the TRFs to local stakeholders. For instance, in Xing’an county, Guangxi Province, the TRF for the rice project in Maiyuan had become more commercialised. PPS-CN outsourced the business operations to an interested local entrepreneur, who was willing to invest funds into the TRF to make it a private sector company. PPS-CN will remain the most important customer, using government funds to purchase biological control products for farmers or demonstration plots, and will also remain the owner of the facility, providing the necessary technical support, but not taking any profits. 

The positive impact of the IPM interventions for both crops is also visible in the farmers’ improved yields. For instance, evidence suggests slightly increased yields, due to the implementation of a biological control and other IPM measures, promoted by the interventions. Even though this yield increase was not statistically significant, the finding is in line with previous experimental outcomes, established during the rice IPM project, where the yields were found to be about 5% higher in the IPM plots in all of the three target countries [11].

Very positive feedback was provided in relation to the introduction of biological control-based IPM in rice and maize from involved farmers, IPM trainers, and extension staff, as well as from higher level officials, including the Department of Agriculture. However, the assessment also suggests that farmers were not seeing enough benefits from the *Trichogramma* releases to persuade them to pay a price for the product, that would enable sustainable production. Farmers in the maize IPM project, as the owners of the TRFs had the chance to distribute the *Trichogramma* egg-cards to their fields, but largely failed to do so. A number of factors likely contributed to this, most importantly, it proved difficult to produce sufficiently high numbers of biological control agent, particularly during peak pest occurrence, while maintaining a high-quality product. 

Another important result of the interventions was that national partners planned to promote the *Trichogramma* cards in some high-value crops, like vegetables, citrus fruits, and grapes, in which farmers were more willing to invest more in agro-inputs. Even though the species reared in the projects might not be the optimal biological control agent for these high-value crops, and a number of other factors need to be adapted (e.g., the release strategy), positive first results were obtained from the release of *Trichogramma* in 13 ha of citrus orchards in Guangxi Province, targeting swallowtails and noctuid moths (PPS-CN, personal communication). Generally, biological control methods are more widely and easily adopted in high-value crops, where net income per ha is more than an order of magnitude higher than when growing rice or maize. Thus, it may be advisable to initially focus on high-value crops when trying to introduce a biological control in the target countries. Furthermore, when an important objective is to reduce pesticide use, crops other than maize or rice—where only small amounts of pesticide are used, at least in Laos and Myanmar—need to be selected.

The study found that farmers who spent more money on pesticides to protect their crops did not have correspondingly better yields, which indicates a reduction in profitability for farmers using lots of pesticides, as pesticides costs are generally higher. Even though in particular cases, higher pesticide applications may have been justified, due to high pest pressure, it indicates that relying on less chemicals through rice or maize IPM may be a more effective choice. It is also in line with studies showing that chemical insecticides may adversely affect pest regulatory ecosystem functions, leading to higher pest damage in the crop (shown for rice by Horgan et al. [23]). Thus, evidence is accumulating that suggests not to spray rice fields with insecticides during the first 40 days after transplanting, again confirmed by a study from China [24].

Altogether, the biological control-based IPM approach, introduced to the target region through the two interventions brought a number of benefits, the reductions in pesticide use and the increase in yields are relatively minor when compared to the analysis of 85 IPM projects from across Asia and Africa [7]. In the projects assessed here, however, farmers’ adoption of IPM techniques was less than anticipated. It is known that, in addition to agronomic aspects, a number of social, political, economic, and ecological factors determine whether or not IPM can work, or not work. Specifically, it has been demonstrated by Morse and Buhler [25] that often, resource-poor farmers do not match the necessary criteria for the implementation of IPM programmes. Factors driving uptake of IPM practices in the two projects were not clear, though it was noted that some of the IPM measures adopted (e.g., use of free *Trichogramma* cards, reduced number of pesticide sprays) were likely to reduce the costs of production for smallholder farmers focused on subsistence farming and local markets. Morse and Buhler [25], and Pingali and Gerpacio [26], observed that labour intensive practices can also be a disincentive, particularly when the cost of labour is high, and the markets, weak. The two measures implemented by farmers (biocontrol and installation of traps) are in fact not time-consuming, and even linked to reduced time needed for applying pesticides, but labour could have been a factor for other IPM measures not being implemented. 

Despite considerable efforts by project partners, individual farmers received a relatively small amount of training on the IPM measures, especially other measures in addition to releasing *Trichogramma* egg-cards. More intensive training, paired with more demonstration fields and more efforts in raising awareness, may increase the adoption of improved practices, as was suggested by virtually all stakeholders during the KIIs. A lack of training in IPM implementation and raising awareness of the benefits of such methods has been noted by Parsa et al. [9] as the primary reason for the failure to implement IPM measures. However, even though information is a necessary pre-requisite for change, appropriate incentives need to be in place first, and these are often not obvious for farmers, particularly smallholder farmers. For instance, Dijkxhoorn et al. [27] observed that, while 70% of export-oriented farmers in East Africa are implementing IPM, smallholders’ adoption rates was minimal. In their explanation for this, Dijkxhoorn et al. [27] refer to low market incentives for farmers to start implementing IPM, as well as a lack of knowledge and financial resources to do so. Thus, IPM interventions need to focus on crops where there are incentives that are likely to facilitate the intended changes, and ensure the necessary financial and information resources are readily available. 

An important outcome of the interventions discussed here is that the national partners in Laos and Myanmar have become national competence centres for biological control, whilst the Chinese partners in Yunnan and Guangxi Provinces have become provincial competence centres for biological control. The combination of enhanced knowledge from international projects and national support, through policy changes towards ‘greener’ agricultural production, allows these partners to research and implement more biological control-based solutions for urgent pest problems in the target GMS countries.

## 5. Conclusions

Biological control-based IPM in rice and maize was a relatively new concept for the target countries in the GMS. Half of the 22 TRFs established by the assessed projects stopped producing and delivering egg-cards to farmers, and government funding support was found to be essential for the continued operation of the TRFs. However, both rice and maize farmers expressed very positive feedback towards biological control-based IPM, but more training, demonstration plots, and awareness creation is needed to increase uptake beyond the relatively small project implementation areas. IPM practices, such as the introduction of biological control agents, would benefit from targeting farmer cooperatives, as well as high value and high yielding crops. By anchoring biological control and several other IPM practices at governmental, extension, and, at a still relatively small-scale, farmer level, the two interventions have helped pave the way for more sustainable maize and rice production, as well as establishing a better environment and healthier lives for the farm households in the target region.

## Figures and Tables

**Table 1:** Locations where *Trichogramma* rearing facilities (TRF) were established (the year when mass *Trichogramma* rearing started); number of treatment (T) and control (C) farmers in household questionnaire surveys (HHS) in Southwestern China, and number of focus group discussions (FGD). On-site visits to implement KIIs were carried out in locations in bold or with individuals from locations in bold italics travelling to a central location.

**SW China-Village/Township**	**Laos-Districts**	**Myanmar Townships**
**Maize**		
Dehong prefecture, Yunnan Province **Tuanqing** (2014) [50T;10C], *2FGD* Husa (2015) **Sudian** (2015) *2FGD*	Vientiane Province **Sanakham (2015)**, *2FGD* Xaiaboury Province Paklai (mass production did not start)	Southern Shan State **Thanlun** (2014) **Pinphit** (2015), *1FGD* Northern Shan State Kharshi (2016) Sakhanthar (2016)
**Rice**		
Dehong prefecture, Yunnan province **Tuanqing** (2014) **Mangshi PPQS** (2014) [51T; 11C], *2FGD* Xing’an county, Guangxi Province ***Maiyuan*** (2014) **Hejiatang** (2015) [54T; 19C], *2FGD*	Vientiane Capitol Province **Vientiane PPC** (2013), *2FGD* Sayabouri Province ***Phieng*** (2015) Sayabouri (2015) Xienghou (2015)	Yangon region **Yangon PPD** (2015), *4FGD* Sagaing region Paleik (2015) Mandalay region ShweBo (2015) Naypyidaw Union Territory Yezin Agric. University (2016)

**Table 2:** Number of key informant interviews conducted in the three target countries for the maize and rice crop (total = 61).

	China		Laos		Myanmar	
	Maize	Rice	Maize	Rice	Maize	Rice
Project implementation team	1	3	1	1	1	1
Local agricultural authorities	0	2	1	2	1	0
Local IPM trainers/extension	3	1	1	2	1	1
TRF managers and staff	3	4	1	2	1	1
Village Committee members	3	2	0	1	2	1
Customers	0	2	0	0	1	3
Agro-input shops	3	4	1	1	1	1
Total	13	18	5	9	8	8

**Table 3:** Situation of TRFs established within the maize and rice integrated pest management (IPM) projects during the last year of the project (mid-2016) and by the end of 2017.

		**No. TRFs Running Mid-2016**	**No. TRFs Running End 2017**	**No. TRFs only Stock Rearing in 2017**	**No. of TRFs Producing *Trichogramma*** **for Release 2017**	**Area treated (ha) with *Trichogramma* in 2017**
**Rice Project**	Laos	4	4	2	2	222
	Myanmar	4	4	0	4	416
	China	4	2	0	2	415
	Sub-total	12	10	2	8	1,053
**Maize Project**	Laos	1	1	0	1	270
	Myanmar	3	1	1	0	0
	China	4	3	1	2	554
	Sub-total	8	5	2	3	824
Sum total		20	15	4	11	1877

**Table 4:** Socio-economic characteristics of the sampled farmers during the Householder Questionnaire Survey (HHS).

	Treatment (n = 155)		Control (n = 40)	
	Mean	SD	Mean	SD
Household size	4.86	1.81	5.08	1.87
Number of household members involved in farming	2.55	1.05	2.45	1.08
Most educated household member had completed high school (%)	23.9		17.5	
Total land owned by household (hectare)	0.37 *	0.28	0.29	0.17
Amount of own land farmed (hectare)	0.36	0.25	0.29	0.16
Amount of rented land farmed (hectare)	0.22	1.35	0.42	2.05
Area under rice production (hectare)	0.36	0.53	0.27	0.19
Area under maize production (hectare)	0.21	0.15	0.21	0.09

Note: for results related specifically to rice, n = 105 and 30 for treatment and control, respectively; and for maize, n = 50 and 10 for treatment and control, respectively. * indicates that the mean values for treatment farmers are significantly different from control farmers, based on *t*-tests, at the 5% significance level.

**Table 5:** Summary statistics of farm-level benefits reported by rice and maize farmers during the HHS in the last cropping season of 2017.

	Treatment		Control	
	Mean	SD	Mean	SD
Quantity of rice harvested (kg)	2194.9	1383.5	1788.3	1280.2
Quantity of maize harvested (kg)	1076.1	939.9	937	682.7
Rice yield (kg/ha)	7213.3	2590.2	6932.3	1328.7
Maize yield (kg/ha)	5011.2	1726.2	4535.0	2200.6
Household applied pesticides to rice (%)	99.1		100	
Household applied pesticides to maize (%)	66.0		70.0	
Amount spent on chemicals for rice (USD/ha)	148.9 *	112.5	201.6	139.9
Amount spent on chemicals for maize (USD/ha)	144.9	110.5	178.6	90.9

Note: for results related specifically to rice, n = 105 and 30 for treatment and control, respectively; and for maize, n = 50 and 10 for treatment and control, respectively. * indicates significant differences between treatment and control farmers, based on *t*-tests, at the 5% significance level.

**Table 6:** Reasons for a decrease in pesticide use reported by rice and maize farmers during the HHS between 2015 and 2017, values given as a percentage.

Reason	Treatment (n = 86)	Control (n = 15)
1. I learnt from project training or other information resources to improve alternative pest control methods	37.2	20.0
2. Control effects of pesticide were better than last year	18.6	6.7
3. Fewer pest and disease outbreaks	17.4 **	53.3
4. Used alternative IPM methods promoted by other projects (e.g., light traps, pheromone traps)	63.9	40.0
5. Applied *Trichogramma* egg-cards	94.2 ***	20.0
6. It is not up to me to make the decision on pesticide use	1.2	6.7
7. The government provided pest control services free of charge	5.8	0
8. Pest control provided twice, free of charge	41.9	46.7

** and *** indicate that there are significant differences between treatment and control farmers, based on *t*-tests, at the 1% and 0.1% significance level, respectively.

**Table 7:** Rice and maize farmers’ reported benefits of using *Trichogramma* egg-cards during the HHS, comparing the situation in 2016 and 2017, i.e., before and after applying egg cards.

	Before Applying Egg-Cards		After Applying Egg-Cards	
	Mean	SD	Mean	SD
No. of insecticide sprays on rice (93)	4.24 ***	1.20	2.23	1.2
No. of insecticide sprays on maize (29)	1.93 ***	1.51	1.14	1.16
Cost of insecticides used on rice (86)	620.3 ***	546.1	386	430.8
Cost of insecticides used on maize (30)	248.3 ***	261.7	153.5	213

Note: The sample size is provided in brackets and costs are given in Chinese Yuan. *** indicates that the mean values before applying egg-cards are significantly different from those after applying egg-cards at the 0.1% significance level, based on *t*-tests.
